# Books and Magazines

**Published:** 1905-10

**Authors:** 


### Books and Magazines.

A Text-Book of the Practice of Medicine.—By James Anders, M. D., Ph. D., LL. D., Professor of Medicine and of Clinical Medicine at the Medico-Chirurgical College, Philadelphia. Seventh edition, revised and enlarged. Octavo of 1297 pages, fully illustrated. Philadelphia and London. W. B. Saunders & Company, 1905. Cloth, $5.50 net; Sheep or Half Morocco, $6.50 net.

A sale of over 22,000 copies and the attainment of a seventh edition seems sufficient recommendation for any book; in fact, Anders' Practice does not now need any recommendation—it is too well known. As in the former editions, particular attention is bestowed upon inductive diagnosis, differential diagnosis, and treatment. Regarding differential diagnosis, we notice with much satisfaction that the many diagnostic tables of simulating diseases have been retained. The clinical value of these tabulated points of distinction is beyond cavil. Numerous new subjects have been introduced, among which are: Rocky Mountain Spotted Fever, Examination of Patients for Diagnosis of Diseases of the Stomach, Splanchnoptosis, Cammidge's Test for Glycerose in the Urine, and Myasthenia Gravis. Certain other individual affections have been entirely rewritten and important additions have been made to the diseases which prevail principally in tropical and subtropical regions. The seventh edition of Dr. Anders' Practice maintains the reputation of the work as the best practice before the profession today.

---

Abdominal Operations.—By B. G. A. Moynihan, M. S. (London), F. R. C. S., Senior Assistant Surgeon to Leeds General Infirmary, England. Octavo of 695 pages, with 250 original illustrations. Philadelphia and London. W. B. Saunders & Company, 1905. Cloth, $7.00 net.

It has been truly said of Mr. Moynihan that in describing details of operations he is at his best. This, his latest work, therefore, will be widely welcomed by the medical profession generally, giving as it does in most clear and exact language the preliminary technic of preparation and sterilization, as well as the actual *modus operandi* of the various abdominal operations. Mr. Moynihan's reputation in this field is international, and this work, stamped with the authority of a rare experience, is undoubtedly to become the recognized standard. Peritonitis and appendicitis, the latter of such present importance, have been accorded unusual space in a work of this kind; and the subject of chronic gastric ulcers is also excellently detailed. Throughout the entire book numerous cases have been quoted from both the author's own practice and those of other distinguished surgeons. The beautiful illustrations are all new and have been drawn especially for Mr. Moynihan's work under his personal supervision. The book is a valuable production and adds greatly to the reputation of its eminent author.

---

Atlas and Epitome of Diseases of the Skin.—By Professor Dr. Franz Mracek, of Vienna. Edited, with additions, by Henry W. Stelwagon, M. D., Professor of Dermatology, Jefferson Medical College, Philadelphia. Second edition, revised, enlarged and entirely reset. With 77 colored lithographic plates, 50 half-tone illustrations, and 272 pages of text. Philadelphia and London. W. B. Saunders & Company, 1905. Cloth, $4.00 net.

It is with much pleasure that we review the second edition of Professor Mracek's admirable hand-atlas. That the work is a success and of practical usefulness needs no further proof than the demand for a second edition, not only in America, but also in Germany. The author has added some twenty-six new plates, fifteen of them colored lithographs, and all of exceptional merit. The text he has thoroughly revised to include the most recent dermatologic advances, especially along the line of histopathology. As in the first edition, there is evidence of the conscientious editorial work of Dr. Stelwagon, many additions being interpersed throughout the text.

---

JACKSON ON THE SKIN.—A Ready Reference Hand-book on Diseases of the Skin. By George Thomas Jackson, M. D., Chief of Clinic and Instructor in Dermatology, College of Physicians and Surgeons (Columbia University), New York. Fifth edition, enlarged and thoroughly revised. In one 12mo volume of 676 pages, with 91 engravings and 3 colored plates. Cloth, $2.75, net. Lea Brothers & Co., Publishers, Philadelphia and New York, 1905.

The great value of this volume lies in the clearness of its symptomatology and diagnosis, and the excellent judgment used in its therapeutic recommendations.

The clear diction and the very convenient alphabetical arrangement renders the work not only an exceedingly quick reference book for the busy physician, but adapts it especially to the needs of students. The demand for five large editions is ample evidence of the popularity of the book. Each edition presents a thorough revision of the subject, so that the work may always be consulted for the condition of the science of Dermatology as it really exists. The present revision has been particularly searching, and the subject matter has been brought well up to date. The appendix, containing formulæ for baths, lotions, ointments, powders, etc., and prescriptions for internal treatment, is alone worth the price of the book.

As heretofore, symptomatology, diagnosis and treatment are specially considered. Many new sections have been added, resulting in a considerable enlargement of the work, and the volume is issued in full confidence that it will prove even more than ever before valuable to practitioners, students and teachers.

---

A Manual of the Practice of Medicine.—By A. A. Stevens, A. M., M. D., Professor of Pathology in the Woman's Medical College of Pennsylvania, and Lecturer on Physical Diagnosis at the University of Pennsylvania. Seventh edition, revised; 12mo of 556 pages, illustrated. Philadelphia and London. W. B. Saunders & Company, 1905. Flexible Leather, $2.50, net.

We know of no work on practice of the same size containing so much practical information concisely stated, as this handy little book by Dr. Stevens. The author's epigrammatic style is no doubt the result of his extensive experience in the lecture room, enabling him to group allied symptoms in such a manner that they can be easily retained in the mind of the student. By a judicious elimination of theories and redundant explanations he has brought within a small compass a complete line of practice of inestimable value. Indeed, for the student, the practitioner, and the nurse as well, we know of none better.

---

A Text-Book of Diseases of Women.—By Barton Cooke Hirst, M. D., Professor of Obstetrics, University of Pennsylvania. Second edition, revised and enlarged. Octavo of 741 pages, with 701 original illustrations, many in colors. Philadelphia and London. W. B. Saunders & Company, 1905. Cloth, $5.00, net; Sheep or Half Morocco, $6.00, net.

Dr. Hirst may well be congratulated upon the publication of such a work as this, a second edition of which has just appeared. Written on the same line as his "Text-Book of Obstetrics," to which it may be called a companion volume, it gives every promise of attaining a similar success. The palliative treatment of diseases of women and such curative treatment as can be carried out by the general practitioner have been given special attention, enabling physicians to treat many of their patients without referring them to a specialist. Indeed, throughout the book great stress has been laid upon diagnosis and treatment, and the section devoted to a detailed description of modern gynecic operations is without doubt the most clear and concise we have yet read. In this second edition the revision has been thorough, introducing, however, only such matter that promises or has been demonstrated to be of permanent value. Forty-seven new illustrations have been added and thirty of the old ones replaced, the work now containing a collection of 701 beautiful original illustrations, many of them in colors. We take much pleasure in recommending Dr. Hirst's work to the medical profession generally.

---

LECTURES UPON THE PRINCIPLES OF SURGERY.—With an Appendix Containing a Resume of the Principal Views held Concerning Inflammation. By Charles B. Nancrede, M. D., LL. D., Professor of Surgery and of Clinical Surgery, University of Michigan, Ann Arbor. Second edition, thoroughly revised. Octavo of 407 pages, illustrated. Philadelphia and London. W. B. Saunders & Company, 1905. Cloth, $2.50, net.

The difficulty with the very great majority of works purporting to treat of the principles of surgery is that they attempt to be too comprehensive, so marring their usefulness to the undergraduate. Dr. Nancrede, whose work is now before us, has studiously aimed to overcome this objection, and has met with unqualified success. His work is not a new one, but for some years has held a place of first importance amongst medical text-books. The appearance of this new second edition, therefore, will be a source of much gratification to those who have found the work so valuable. Much has been added regarding the significance of leukocytosis, the treatment of sepsis and of tetanus, and the after-effects of general anesthesia and spinal anesthesia. By rewriting some portions and by greatly modifying others, Dr. Nancrede has brought his excellent work to a degree of perfection only attainable by the careful observation and study of a rich clinical experience.

